# Community-driven strategies for preventing childhood stunting: perspectives from parents and front-line workers in Rwanda

**DOI:** 10.3389/fnut.2026.1725510

**Published:** 2026-02-19

**Authors:** Jean de Dieu Habimana, Theogene Habumugisha, Eric Matsiko, Joseph Karemera, Noel Korukire, Maryse Umugwaneza, Lawrence Rugema, Cyprien Munyanshongore

**Affiliations:** 1 School of Public Health, College of Medicine and Health Sciences, University of Rwanda, Kigali, Rwanda; 2 Centre for International Health, Department of Global Public Health and Primary Care, University of Bergen, Bergen, Norway; 3 Rwanda Food and Drug Authority, Kigali, Rwanda

**Keywords:** local leaders, childhood stunting, community health workers, community-driven strategies, farmer promoters, front-line workers, Inshuti z’ Umuryango, parents

## Abstract

**Background:**

Childhood stunting remains a major public health challenge in Rwanda, affecting child growth, development, and long-term health outcomes. This study explored the views of parents and front-line workers to inform community-driven strategies to prevent stunting.

**Methods:**

Focus Group Discussions (FGD) and Key Informant Interviews (KII) were conducted among 83 parents and front-line workers in five districts of Rwanda, including Nyaruguru, Rutsiro, Burera, Kayonza, and Gasabo, all purposively selected based on their high prevalence of stunting in their respective provinces and Kigali City. The conventional content analysis approach was used to identify the main themes.

**Results:**

Three core themes were identified from the data and were related to: (1) perceived roles of community-led nutrition and hygiene interventions, (2) challenges in the implementation of community-based programmes, and (3) suggested approaches to enhance community participation and adherence. The highlighted strategies included strengthening caregiver capacity, promoting nutrition education, improving access to health and family planning services, and supporting economically feasible interventions such as facilitating access to fruits through fruit trees and small livestock growing.

**Conclusion:**

Enhancing community coordination, promoting nutrition education, ensuring access to health and family planning services, and strengthening caregiver capacity were suggested as essential community-driven strategies to reduce stunting.

## 1 Introduction

Childhood stunting remains a persistent public health concern in many low- and middle-income countries, particularly in sub-Saharan Africa. Globally, approximately 23.2% of children under 5 years of age are stunted, with the highest burden concentrated in Africa and South Asia (1). Rwanda continues to face this challenge despite substantial progress in expanding maternal and child health services. Recent findings of the Rwanda Demographic and Health Survey indicate that about one-third of children under 5 years of age are stunted (2–4).

Stunting, which is a chronic form of undernutrition, reflects long-term nutritional deprivation and exposes affected children to lasting cognitive, health, and economic disadvantages (1). It arises from multiple interlinked factors, including household poverty, food insecurity, maternal undernutrition, poor sanitation, and restricted access to quality health services (5–8). These determinants interact with contextual vulnerabilities such as low parental education, rural–urban disparities, limited household resources, and infrastructural gaps in remote communities (5–7). Furthermore, inadequate infant and young child feeding practices, limited knowledge of hygiene and sanitation, and suboptimal acceptance of preventive health services often arise from these broader constraints and reinforce the cycle of undernutrition (8, 9).

In Rwanda, studies indicate that stunting is mainly driven by modifiable factors, including poverty-related food insecurity, inadequate dietary diversity, poor sanitation and hygiene, limited access to safe water, inadequate use of maternal and child health services (including antenatal care), suboptimal infant and young child feeding practices, and low maternal education (10, 11). These risk factors tend to cluster in rural and socioeconomically disadvantaged households, where limited infrastructure, lower educational opportunities, and restricted access to health and nutrition services further exacerbate children’s vulnerability to chronic undernutrition (9). Together, these interacting conditions create an environment in which children are more likely to experience sustained nutritional deprivation, ultimately leading to impaired growth and development (12).

To combat these challenges, the Government of Rwanda has implemented various nutrition-specific and nutrition-sensitive programmes. These include Early Childhood Development Centres (ECD), kitchen gardens, school feeding initiatives, the Girinka ‘One Cow per Poor Household” programme, and community volunteers who provide nutrition and health education at the community level (13, 14). Although these efforts have contributed to national progress, stunting levels remain high with 33.8% in 2020 (2, 4, 6). Evidence shows that interventions that leverage local actors, social capital, and community ownership achieve greater sustainability and impact in the resolution of nutritional problems (15).

In Rwanda, front-line workers include Community Health Workers (CHWs), farmer promoters, Friends of the Family (Inshuti z ‘Umuryango: IZU), and local leaders play a critical role in bridge the gap between household practices and national policies (14). Their influence goes beyond information dissemination; they shape daily behaviour, monitor vulnerable households, and support families in adopting recommended health and nutrition practices. However, parents remain the primary decision-makers regarding feeding, hygiene, and childcare, making their experiences and challenges central to the success of any stunting prevention strategy.

Although parents and front-line workers in the community have been shown to play a crucial role in influencing child nutrition and care practices, their views on stunting prevention have not been fully documented, including those of Rwanda. Most studies to date have been conducted with an epidemiological focus on the determinants of stunting and the efficacy of interventions, while little is known about how community actors construct and implement stunting prevention activities in their daily practice. This study complements previous research efforts that have focused predominantly on epidemiological determinants of stunting, providing in-depth qualitative insights into how parents and front-line workers understand, prioritise, and act on the prevention within Rwanda’s distinctive community governance systems and volunteer structures. Therefore, this article examines the perspectives of parents and front-line workers to guide community-based interventions for the prevention of childhood stunting in Rwanda.

## 2 Methods

### 2.1 Study design and setting

This qualitative phenomenological study used focus group discussions (FGD) and key informant interviews (KII) in five districts distributed from the five provinces of Rwanda. These five districts (Kayonza, Nyaruguru, Burera, Rutsiro, and Gasabo) were purposively selected based on their high prevalence of childhood stunting in each of the five provinces of Rwanda, namely the Eastern Province, Southern Province, Northern Province, Western Province, and Kigali City, respectively (16).

### 2.2 Study participants

Eighty-three participants were purposively recruited to participate in the study, including 38 parents and 45 front-line workers. The parents consisted of mothers and fathers, and the selection criteria were based on being classified in low-income households (based on Ubudehe Classification) (17), and having both stunted and non-stunted children aged 6 to 23 months. The identification of parents was done through the CHWs’ register network. Front-line workers consisted of community health workers (CHW), Friends of Families (IZU), Community Farmer Promoters, and people in charge of Social Affairs at Village level. In addition to these community volunteers, Health Centre Managers and a Nutrition Officer were conveniently selected from a Health Centre in each of the sampled districts. An early childhood promoter was also recruited from each district.

### 2.3 Data collection procedures

A total of 10 FGDs were conducted among parents and Community volunteers, including CHWs, *IZU*, Community Farmer Promoters, and Village Social Affairs representatives. Sixteen KIIs were also conducted for Health Centre Managers, Nutrition Officers, and ECD Promoters. A pilot test was conducted before data collection. This pilot helped to familiarise themselves with the data collection tools, check their clarity, and confirm that they addressed the objectives of the study. All FGDs and KIIs used open-ended questions designed to elicit participants’ perceptions of childhood stunting. Furthermore, the lead investigator facilitated all interviews together with a research assistant who recorded and documented detailed notes. All interviews were conducted in Kinyarwanda, with sessions lasting between 40 and 80 min. After each data collection, the study team analysed audio recordings and field notes to clarify misunderstandings and highlight significant observations. Data collection continued until saturation was achieved, defined as the point at which no additional information, perspectives, or analytic categories emerged from subsequent FGD or KIIs.

### 2.4 Data processing and synthesis

Prior to analysis, all audio recordings were transcribed verbatim in Kinyarwanda (the original language) and later translated into English by qualified bilingual translators. Two bilingual experts conducted an independent reverse translation to verify accuracy and ensure that the original meaning was preserved (18). The transcripts were also reread to familiarise with the data (19).

**Figure 1:**
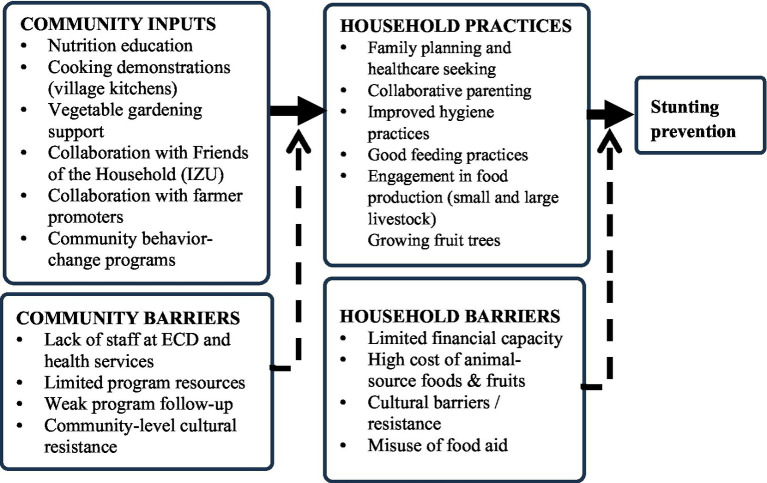
Conceptual framework derived from participant perspectives on stunting prevention.

Conventional Content Analysis was used to synthesise the data. This approach is appropriate for studies that seek to extract meaning directly from participant narratives without imposing previous theoretical frameworks (20). The inductive coding approach to coding was used to code the data set, and the open coding process was performed independently by the primary investigator and one coinvestigator. During the analysis, significant textual passages were highlighted and labelled with descriptive terms that reflect the experiences and expressions of the participants in two focus groups and two key informants (21) (Figure 1). The initial codes were shared and discussed by the group of authors. After discussion, the codes were refined and placed on the list for the codebook. Reflective memos were used throughout the analysis to document analytical decisions and researcher reflexivity. The research team consisted of public health researchers with experience in nutrition and community health in Rwanda. Reflexive memos were used to acknowledge and minimise the influence of prior assumptions of researchers and the professional backgrounds of researchers on data interpretation.

The emerging subthemes identified from the data were combined into more general subtheme categories that reflected the participants’ perceptions of community-driven stunting prevention strategies (22). Several techniques were used to increase the robustness of the analysis, including code agreement, peer debriefing, and member validation with three participants to validate interpretations (23). Data were managed and organised using NVivo software (version 14).

## 3 Results

Three core themes were identified from the data (Table 1). The first theme was related to the perceived roles of community-led nutrition and hygiene interventions, and it encompassed three sub-theme categories, including the role of vegetable gardening, the role of village kitchen demonstrations, and the role of nutrition education.

**Table 1:** Core themes, sub-themes, and sub-theme categories of the study.

Core themes	Sub-theme	Illustrative category
Perceived roles of community-led nutrition and hygiene interventions	Role of local nutrition programmes	Role of vegetable gardening
		Role of village kitchens demonstration
	Role of behavioural change programmes	Role of Nutrition Education
Perceived Challenges in the Implementation of Community-Based Interventions	Resource and Economic Barriers	Limited financial support for households
		Misuse of Food Aid
		Lack of sufficient staff in Early Childhood Development (ECD) programmes and other health services
		Elevated cost of animal source food and fruits
	Social and Cultural Resistance	Poor understanding and resistance among households
		Cultural and behavioural barriers
Suggested strategies to enhance community engagement and programme adherence to Stunting Prevention	Strengthening Collaboration front-line workers/community volunteers.	Enhancing collaboration with CHWs
		Enhancing collaboration with Friends of the Family (IZU)
		Enhancing collaboration with community farmer promoters
	Compliance with health advice	Family planning and health care seeking
		Collaborative parenting
		Improving hygiene practice
	Adherence to food production programmes	Promoting a large and small livestock programme
		The growing fruit trees in all public places

The second theme consists of the perceived Challenges in Implementation of Community-Based Interventions, and it included six sub-theme categories, including limited financial support for households, misuse of food aid, inadequate staffing in Early Childhood Development (ECD) programmes and other health services, excessive cost of animal-source foods and fruits, poor understanding and resistance among households, and cultural or behavioural barriers.

**Figure 2:**
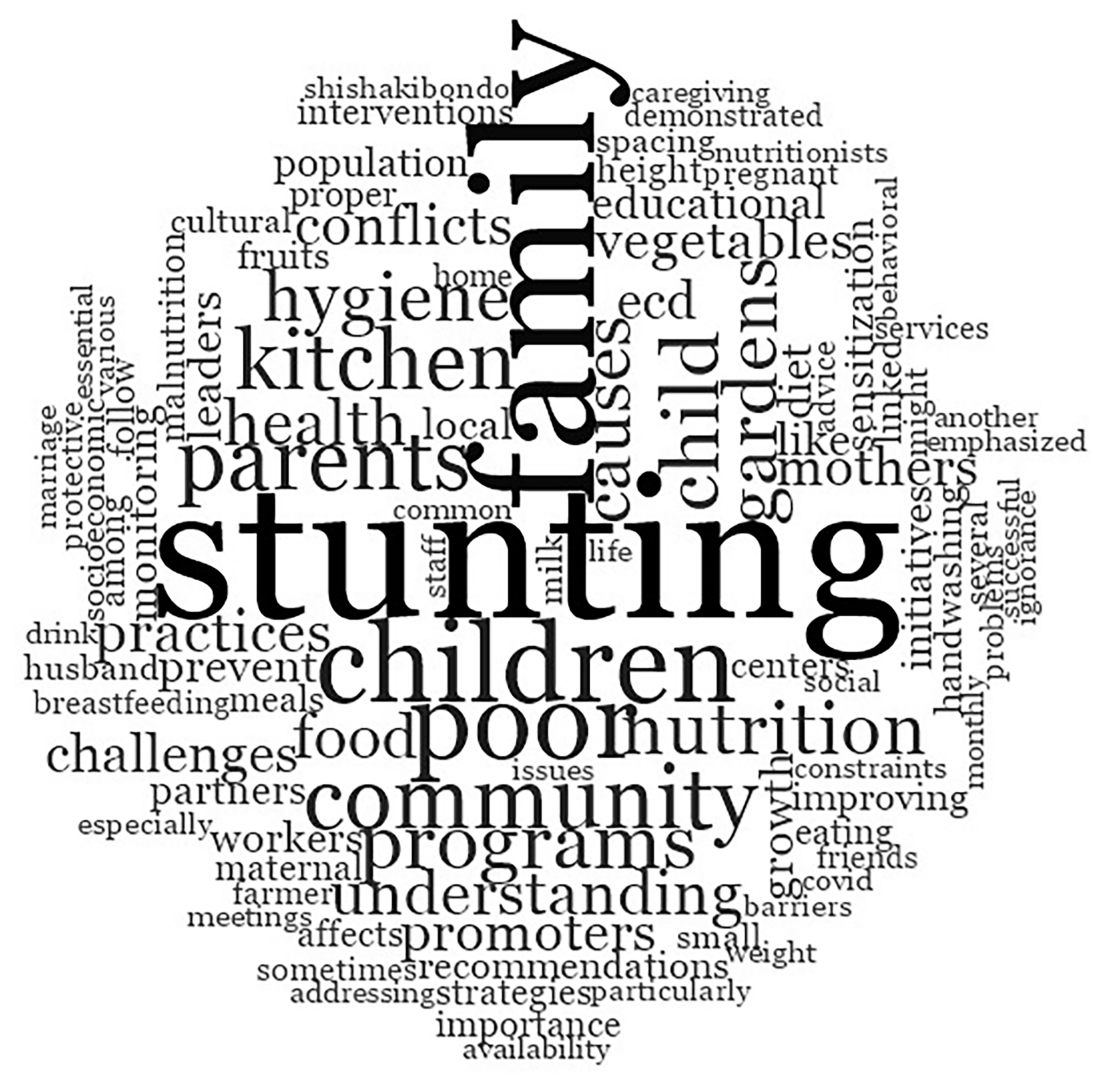
Key qualitative themes.

The third theme evolved around strategies to enhance community engagement and programme adherence for stunting prevention and comprised several strategies including enhancing collaboration with local leaders, CHWs, Friends of the Families and Community Farmer, promote family planning and health care seeking, collaborative parenting, improving hygiene practice, Good feeding practice; supporting both small and large livestock programmes; and promoting fruit tree planting in public spaces (Figure 2).

### 3.1 Detailed views expressed by study participants from FGD and KII

#### 3.1.1 Theme 1: perceived role of community-led nutrition and hygiene interventions

##### 3.1.1.1 Role of vegetable gardening

The findings from the analysis highlighted the positive influence of agricultural extension in the home, when aligned with nutritional education, on household dietary practices. A parent explained how kitchen gardens contribute: *“Households that have kitchen gardens can avoid stunting because vegetables are essential for balanced meals. When you grow them at home, you do not need to worry about buying them from the market every day.”* (Parent 3, District 4).

##### 3.1.1.2 Role of the village kitchen demonstration

The participants emphasised the importance of organised community-based initiatives in the ongoing promotion and monitoring of children’s health. The child growth monitoring programme, which is integrated into the village kitchen programme, plays a significant role in promoting early detection of childhood malnutrition and teaching parents how to prepare a balanced diet. The participants underscored the crucial role of CHWs in these initiatives by conducting monthly growth monitoring activities at the village level. An ECD promoter in one District said: *“There is a national village kitchen programme and a programme to follow the growth of children where CHWs check the monthly weight of the children and parents learn how to prepare a balanced diet. These programmes ensure that children’s health is closely monitored.”* (ECD Promoter, District 3).

##### 3.1.1.3 Role of nutrition education

The impact of interventions that target the development of nutrition habits and knowledge of caregivers’ children was a growing concern. An ECD promoter highlighted the importance of GIKURIRO and ORORA WIHAZE, community-based initiatives to increase dietary diversity and educate people about foods rich in nutrients. These programmes were repeatedly mentioned as important contributors to nutritional education. These initiatives focus on encouraging the inclusion of vegetables, animal-based foods, and other vital food groups in daily meals, in addition to educating young children about their nutritional needs. “*We have projects in our District like GIKURIRO teaching healthy food to children and ORORA WIHAZE raising awareness of eating of animal products. These projects help households understand the importance of diverse and nutritious diets.”* (ECD promoter, District 3). In addition, ongoing education campaigns and programmes are also critical. For example, an ECD promoter emphasised the importance of continuous teaching and sensitisation. *“We continue to teach the population how to use what we have, the hand-washing culture. These elements ensure better child health and reduced stunting.”* (ECD promoter, District 3).

#### 3.1.2 Theme 2: perceived challenges in the implementation of community-based interventions

The participants highlighted the enormous demands of daily life and how their capacity to participate in community meetings or health-related initiatives is limited by informal, precarious employment. *“We do not have permanent jobs, we work for money every day, no time for those meetings that do not bring anything. What we need is to get what to feed our children.”* (Parent 6, District 5).

##### 3.1.2.1 Misuse of food aid

The mismanagement of resources provided by the government was also highlighted as a challenge. *“Some parents sell the products given to children by the government, like shishakibondo, saying they need the money for other things. Others use it to buy sorghum beer instead of feeding their children.”* (ECD promoter, District 1).

This misuse reflects the competing priorities that households face due to poverty, further affecting efforts to combat stunting. In addition, sharing food aid among all household members was described as a problem that can hinder child development. “*Parents who receive food aid meant for their malnourished children often share it with the entire household, which can reduce the intended nutritional benefits for the child*.” (Head of Health Centre, District 4).

##### 3.1.2.2 Lack of sufficient staff in early childhood development programmes and other health services

The lack of access to essential services, such as family planning and ECD programmes, was perceived to be a significant barrier. The lack of sufficient staff in the health centres also hindered the ability to sustain ongoing interventions and initiatives. *“No, the ECD staff and nurses are not sufficient.”* (Parent 3, District 4).

##### 3.1.2.3 Elevated cost of animal-source food and fruits

Regarding food costs, participants emphasised that high food prices, especially fruits and items derived from animals, restrict access to many households. “Animal sources of food *are often expensive, and raising cattle is not feasible for everyone”* (Nutritionist, District 3).

*“Fruits are expensive and not available everywhere.”* (ECD Promoter, District 2).

##### 3.1.2.4 Poor understanding and resistance among households

Resistance to change between households creates significant barriers to addressing stunting. Many respondents said that some households are reluctant to follow the advice to change their behaviour and practices. *“You teach them, but they do not put into practice what they learnt. You tell them how important balanced diets and vegetables are, but you go back and find that they are still doing the same thing as before, and the child remains stunted”* (ECD promoter, District 2).

##### 3.1.2.5 Cultural and behavioural barriers

Cultural misconceptions such as believing in witchcraft were also cited as hindering the normal growth of children. One participant, instead of following professional advice, used to rely on peer-generated misinformation. *“Initially, they told me that it is his heart that hinders him from growing, when I inquired about it by others, they told me that it was a witchcraft attack.”* (Parent 4, District 5) Some religious restrictions also were accused of being a barrier to better nutrition: *“Adventists do not eat meat or seafood, which have important nutrients for the baby. This cultural restriction can lead to deficiencies.”* (Parent 8, District 3).

#### 3.1.3 Theme 3: suggested strategies to improve community engagement and adherence to the programme for stunting prevention

##### 3.1.3.1 Enhancing collaboration with community health workers

It was highlighted that CHWs played an essential role in the successful implementation of prevention programmes and education of households. A respondent praised the efforts of CHWs: *“The reason why CHWs prevent children from stunting is that if you follow their advice and have the capacity to take good care of the child, the child cannot be stunted. Their guidance makes all the difference.”* (Parent 8, District 5).

##### 3.1.3.2 Enhancing collaboration with friends of families for conflict resolution

Household harmony and conflict resolution were also considered essential for improving caregiving practices and preventing stunting. It was expressed that Friends of Families play a significant role in fostering peaceful households. “*Friends of Families come to you and advise you in case you have conflicts. They reconcile you and the security returns home which is important to prevent stunting.”* (Parent 7, District 4).

##### 3.1.3.3 Enhancing collaboration with community farmer promoters

The participants emphasised the assistance of local farmers who helped promote the kitchen garden. As a community participant said: “*Community farmers promoters like to visit people who have land to cultivate and give them advice on what to plant, plants with vitamins such as vegetables. This helps households improve their nutrition and combat stunting.”* (Parent 3, District 1).

##### 3.1.3.4 Family planning and health care seeking

Participants from both sides, parents and front-line workers, accentuated the role of family planning and health care-seeking in preventing childhood stunting. *“The secret behind stunting prevention is linked to having fewer children because they understand the benefits of family planning. This allows them to focus on the children they have.”* (ECD promoter, District 3).

Another participant clarified the health care search: *“Usually, stunting starts with conception*. *Therefore, if you go to the health facility as soon as you know that you are pregnant, they will give you advice on what to eat and what to avoid protecting your baby.”* (Parent 4, District 5).

##### 3.1.3.5 Collaborative parenting

Like previously, both parties underscored the role of family planning and harmonious relationships as crucial protective factors against childhood stunting. Some CHWs explained the importance of shared goals in a household: *“Households that have never experienced stunting are those who live in harmony, share what they get, and have a common goal like taking good care of their child. When both parents collaborate, the child benefits.”* (CHW1, District 2). Furthermore, the participation of husbands in care was also echoed: “*When their husband is knowledgeable, he might remember that you left a child at home and bring fruit or eggs to that child, which would protect them from stunting.”* (Parent 6, District 5).

##### 3.1.3.6 Improving hygiene practices

Households who adhered to health advice and maintained good hygiene practices were more resistant to stunting. A parent explained the role of hygiene in preventing diseases: *“Even if you have only a few vegetables but they are washed before giving them to the child, you feed them many times, which will protect them from stunting.”* (Parent 7, District 4).

##### 3.1.3.7 Promoting large and small livestock programmes

Livestock ownership was repeatedly pointed out by both parents and front-line workers as crucial in reducing childhood stunting. One parent stated the importance of milk, another mentioned that livestock help in obtaining manure: “*having a milking cow makes a big difference in child nutrition. Households with cows can provide milk regularly, ensuring that children are healthy*.” (Parent 4, District 2) “*You can advocate for us to receive goats to help with farming and fight stunting by improving nutrition*.” (Parent 2, District 4).

Another participant suggested that the programme to help the population grow small livestock could solve the problem of food scarcity from animal sources. *“Small livestock, such as chickens, goats, or rabbits, are more affordable and manageable for most households.”* (Nutritionist, District 3).

##### 3.1.3.8 Providing fruit plants in all public places

The participants also underlined the promotion of planting fruit trees as a strategy: *“One of the most sustainable solutions is to plant as many fruit trees as possible in all public spaces. Planting as many fruit trees as possible in all public spaces is one of the most sustainable solutions.”* (ECD Promoter, District 3; Figure 1).

## 4 Discussion

**Figure 3:**
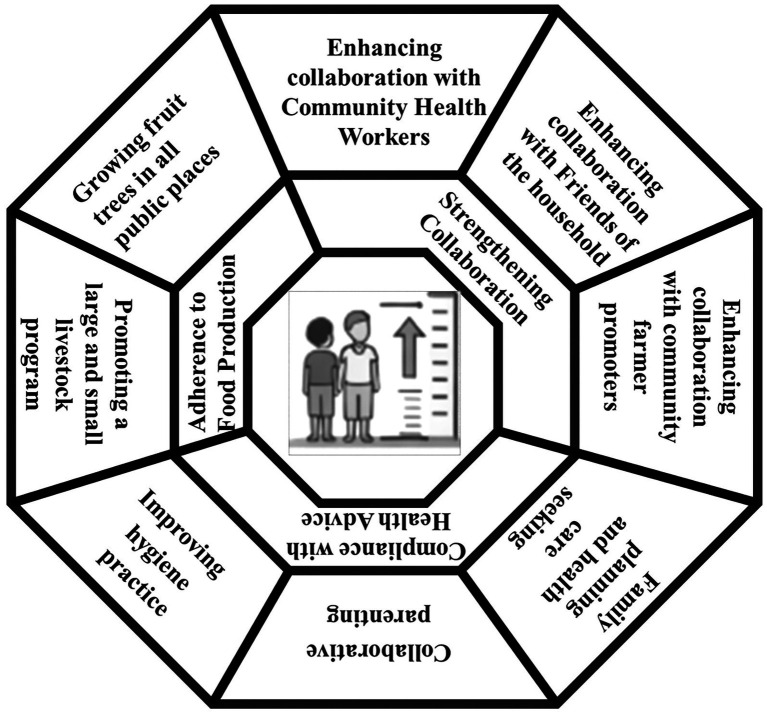
Perceived community-driven strategies for reducing stunting.

This study explored the views of parents and front-line workers to inform community-driven strategies to prevent stunting in Rwanda. Three themes were identified from the data, including perceived roles of community-led nutrition and hygiene interventions, challenges in the implementation of community-based programme strategies, and a set of approaches to improve community engagement and adherence. Potential strategies that were highlighted to prevent stunting included strengthening caregiver capacity, promoting nutrition education, improving access to health and family planning services, and supporting economically feasible interventions (Figure 3).

### 4.1 Perceived role of community-led nutrition and hygiene interventions

Participants perceived community-led nutrition and hygiene interventions as one of the strategies that can be used to eradicate stunting. It was repeatedly highlighted that home-based agricultural extension, combined with nutrition education, positively influenced household diet practices. These findings support the concept of a nutrition-sensitive agriculture approach, which highlights the positive association between food production and better nutrition outcomes (24). In sub-Saharan Africa, these types of integrated initiatives have been shown to improve micronutrient consumption and dietary diversification (25). One of such examples is vegetable gardening, which has been shown to increase productivity and food diversity (26).

The study found that the village kitchen plays a key role in improving caregiver knowledge and skills regarding the preparation of balanced diets using locally available foods. Village kitchen demonstration sessions are considered an important arena for healthier eating habits through exchange of experiences. Indeed, the role of village kitchen demonstration in improving nutrition has been documented in rural areas of Laos, Singapore, and the United States (27–29). In addition to sharing experience, village kitchen demonstration sessions also provide opportunities for parents to reflect on the growth and development of their children. Embedding these activities in village kitchen demonstration sessions has been shown to improve caregiver awareness and promote earlier health-seeking behaviour (27, 30), which in turn positively impacts child health.

The study reported that nutritional education was a vital component in preventing childhood stunting. Nutrition education is provided by CHWs and contributes to increasing the accessibility to culturally relevant nutrition information (31, 32). In addition to accessing information, this component improves access to nutritional advice and counselling, including counselling on feeding infants and young children (33). Reliable access to nutritional information and counselling has been shown to be associated with improved childhood nutritional outcomes in resource-poor settings where this access is restricted (34). Access to reliable nutrition information (from CHW) improves child’s nutrition through improved caregiver feeding practices (35) (Figure 2).

### 4.2 Perceived challenges in the implementation of community-based interventions

This article finds that the lack of formal and secure employment was one of the barriers to better nutrition. In previous studies, limited access to employment has been associated with undernutrition due to poverty and economic uncertainties (11). Additionally, uncertain employment was described to restrict caregivers from participating in local forums that were perceived as beneficial to the nutrition of the child. Therefore, investing in safety net policies that promote the caregiver’s economic certainty can contribute to improved child’s nutrition.

The findings of this study found that the inefficient use of nutrition assistance was also perceived to contribute to childhood stunting in Rwanda. Specifically, it was perceived that the support provided by the government to combat malnutrition was not used effectively. The misuse of nutrition assistance has been reported, where some food insecure households tend to sell or trade food assistance in exchange for urgent needs from other households, such as less expensive staple foods and essential non-food products (36). Similar challenges have been documented in community nutrition programmes, where accountability and oversight reduced the effectiveness of the programme (37–39). This misuse of nutrition assistance prevents nutritional programmes from reducing childhood stunting (40). Therefore, strengthening transparency and community participation in resource management could improve both trust and overall success of government-led initiatives.

The findings showed that limited access to essential services, such as family planning and ECD programmes, was another significant barrier to achieving optimal linear growth. These findings are consistent with previous studies that reported that the absence or underuse of these services can contribute to increased vulnerability to stunting and delayed developmental milestones (41). Limited use of family planning services can lead to closely spaced pregnancies, reducing caregivers’ capacity to provide optimal care. Similarly, limited access to ECD restricts children’s exposure to early stimulation, growth monitoring, and nutrition support, all critical during the first 1,000 days (42). These care gaps can directly affect linear growth and increase vulnerability to stunting, while also affecting cognitive and psychosocial development (43).

The findings of this study underlined the inability to afford food due to unaffordable prices, especially for fruits and items derived from animals. These foods, especially animal-based foods, are nutrient rich and their absence in the diet contributes to growth retardation. Previous studies have shown that food affordability is a significant factor that affects diet decisions and often forces households to rely on less expensive, less nutrient-dense staples (44). Insufficient intake of fruits, vegetables, and animal products can lower the intake of high quality proteins and vital micronutrients, both of which are necessary for proper linear growth (45, 46). These results highlight the importance of interventions that increase food affordability or offer targeted assistance to households at risk, reaffirming that financial limitations are a major factor in determining the nutritional and growth outcomes of children. Price constraints illustrate how broader market dynamics can limit the ability of households to act on nutrition knowledge, even when community support mechanisms are in place (47).

Poor understanding and resistance among households were perceived as potential contributors to childhood stunting. Caregivers who do not believe that certain dietary practices align with their beliefs are less likely to adopt and implement these practices, even in the presence of resources and supervision. Evidence-based guidelines and traditional beliefs and practices about childcare, feeding, and health could clash, causing caregivers to take actions that could harm a child’s development and nutrition (48). Quality of care can be affected by these misunderstandings, as they can restrict participation in health and early development programmes, acceptance of different diets, or appropriate feeding techniques (49, 50).

### 4.3 Suggested strategies to improve community engagement and programme in stunting prevention

The third theme identified in this study was related to the strategies perceived by caregivers, parents, and front-line workers to enhance community involvement in the prevention of stunting. There are no directly comparable findings in Rwandan. However, a study conducted in Ethiopia has shown that the initiatives focusing on caregiver education and active participation in the community are positively associated with child feeding and care practices (51). Furthermore, evidence from Indonesia also shows that when communities work together through coordinated efforts, combining nutrition education, caregiving support, and social mobilisation, meaningful reductions in childhood stunting can be achieved (52). Together, these experiences support the relevance and potential value of community-centred strategies identified in the present study.

The present study found that community volunteers, including local leaders, CHWs, friends of the family, and community farmer promoters, were perceived to be major players in coordinating educational and preventive programmes for stunting prevention. Local leaders, including village heads and grassroots opinion leaders, significantly influence household practices and community norms (53). Their participation helps facilitate the acceptance and adoption of national nutrition policies, strengthens awareness campaigns, promotes appropriate infant and young child feeding practices, and mobilises community resources for nutrition interventions (54, 55). Furthermore, CHWs provide essential health education and monitor child growth, promote contraception, and guide households to adopt appropriate hygiene, nutrition, and health-seeking behaviours while ensuring compliance with recommended practices (56). In addition, IZU, as trusted community members, are essential in supporting conflict resolution, collaborative parenting, and adherence to nutrition and hygiene practices, thus fostering a stable environment for child growth.

The results of this study showed that households that followed healthy behaviour and practices, including family planning, parental collaboration, practicing better hygiene, and proper feeding practices, were perceived to be less likely to have stunted children. Adherence to family planning was also highlighted as an important practice that can contribute to the prevention of childhood stunting. Lack of adherence to contraception increases the risk of unplanned pregnancies, which can compromise maternal health and limit the caregiver’s ability to provide adequate attention and care to each child (57).

Health-seeking behaviours such as attendance at antenatal care, vaccination uptake, growth monitoring, and timely care seeking for childhood diseases are widely examined in the literature as factors associated with child health and nutritional outcomes. Evidence from previous studies suggests that participation in maternal and child health services is related to improved monitoring of child growth and disease management, both of which are relevant to child nutritional status (58, 59). On the other hand, inadequate or delayed health care can exacerbate vulnerability to infections, lack of nutrition, and compromise linear growth (60).

The results of this study show that a larger family can create financial stress, leading to insufficient resources for food, health care, and other essentials necessary for child growth (61). These results are supported by previous studies that showed that lack of family planning can increase the risk of inadequate nutrition, poor hygiene, and reduced access to healthcare, all of which are known contributors to stunting (62). On the other hand, proper family planning allows parents to space births, allocate resources more effectively, and maintain better health and nutrition for both mother and child, thus reducing the probability of stunting (63). Furthermore, the present study found that cooperative household relationships are an important protective factor against stunting.

It was emphasised that when harmony and mutual understanding prevail within the family, key decisions such as family planning, major purchases, food allocation, and savings are made jointly and more effectively. This reinforces the view that active involvement in childcare, including decision making in food, hygiene and healthcare, not only supports mothers, but also fosters a collaborative household environment that promotes optimal child growth (64, 65). This participation encourages the adherence to recommended nutrition and health practices and reduces household conflict, creating a stable, supportive environment that improves the effectiveness of community-based interventions and household-level initiatives aimed at reducing stunting (66, 67).

The present study found that continued observation of adequate hygiene practices is crucial in the prevention of stunting. Similar associations have been reported in studies conducted in Kenya (68). Observing good hygiene practices contributes to the reduction of intestinal infections and diarrhoea diseases that compromise the intestinal microvilli and cause environmental enteropathy, which in turn reduces nutrient absorption and leads to chronic malnutrition (69).

This study found that small livestock ownership was perceived as an important strategy that can be used to combat childhood stunting in Rwanda. The small livestock rearing can help households to access nutrients dense foods (meat, milk, and eggs) and high-quality nutrients, such as protein and essential micronutrients, that are essential for growth and development (70). In addition to nutrition, livestock provide household income, thus increasing purchasing power for expensive and nutritious foods (71). From a production point of view, livestock produce manure, which is also used to increase the production of household foods, including vegetables and fruits (72).

The cultivation of fruit trees in all accessible places, including homes and public places, has been identified in the literature as a strategy associated with improved access to fruits and increased dietary diversity among children. Increased availability of fruits at the household level can contribute to more diverse complementary feeding practices, which are recognised determinants of child nutritional status (73). In addition, homegrown fruits can reduce dependence on market purchases, potentially alleviating financial restrictions that can limit access to nutrient rich foods (74).

## 5 Strengths and limitations

A major strength of the present study was the use of different data collection techniques (FGD and KIIs) and various categories of study participants (caregivers, early childhood workers, CHW and healthcare professionals). Our sampling strategy covered all provinces of Rwanda, increasing representation of views. The use of qualitative design allowed the exploration and capture of issues that may not be identified by quantitative study design. However, the study also presents some limitations. Social desirability bias might have affected interviews, especially given the awareness of some topics such as caregiving, feeding, and conflicts. Furthermore, the analysis did not employ data triangulation with alternative sources, which could limit the verification and depth of the findings.

### 5.1 Implications for research and policy

Despite limitations, the findings of this study provide an important contribution with relevant implications for both research and policy on reducing childhood stunting in Rwanda. The findings of this study highlighted factors that are perceived to restrict efforts to reduce stunting, including the misuse of nutritional assistance, limited access to reliable nutrition information, and the affordability of nutritious foods, such as fruits and animal-based foods. Therefore, future studies are needed to investigate the impact of these factors.

## 6 Conclusion

Strengthening caregiver capacity, promoting nutrition education, ensuring access to health and family planning services, improving coordination among local leaders, health workers, and households, and facilitating economically feasible interventions such as home fruit tree cultivation and small livestock rearing emerged as practical and sustainable approaches to reduce stunting. These findings underscore the need for stronger community-focused nutrition policies and more research on how locally tailored stunting prevention approaches can be expanded and sustained over time (75).

## Data Availability

The raw data supporting the conclusions of this article will be made available by the authors, without undue reservation.
